# Differential effects of spinal motor neuron-derived and skeletal muscle-derived Rspo2 on acetylcholine receptor clustering at the neuromuscular junction

**DOI:** 10.1038/s41598-018-31949-7

**Published:** 2018-09-11

**Authors:** Jin Li, Mikako Ito, Bisei Ohkawara, Akio Masuda, Kinji Ohno

**Affiliations:** Division of Neurogenetics, Center for Neurological Diseases and Cancer, Nagoya University Graduate School of Medicine, Nagoya, Japan

## Abstract

We recently reported that R-spondin 2 (Rspo2), a secreted activator of Wnt/β-catenin signaling, promotes acetylcholine receptor (AChR) clustering and neuromuscular junction (NMJ) formation via its receptor, Lgr5. Rspo2 is expressed highly in spinal motor neurons (SMNs) and marginally in the skeletal muscle, but the origin of Rspo2 at the NMJ remains elusive. We rescued *Rspo2*-deficient (*Rspo2*^−/−^) mice by specifically expressing Rspo2 in the skeletal muscle and SMNs. SMN-specific Rspo2 mitigated or over-corrected abnormal features of the NMJs and AChR clusters observed in *Rspo2*^−/−^ mice including (i) abnormal broadening of enlarged AChR clusters, (ii) three of six abnormal ultrastructural features, and (iii) abnormal expression of nine genes in SMNs and the diaphragm. In contrast, muscle-specific Rspo2 normalized all six abnormal ultrastructural features, but it had no effect on AChR clustering and NMJ formation at the light microscopy level or on abnormal gene expression in SMNs and the diaphragm. These results suggest that SMN-derived Rspo2 plays a major role in AChR clustering and NMJ formation in the postsynaptic region, and muscle-derived Rspo2 also plays a substantial role in juxtaposition of the active zones and synaptic folds.

## Introduction

The release of the neurotransmitter acetylcholine (ACh) from the axon terminal of the spinal motor neuron (SMN) activates the postsynaptic acetylcholine receptor (AChR) and elicits an action potential in the target muscle fiber^1–3^. To ensure efficient neuromuscular signal transmission, dense AChR clusters must be compactly formed against presynaptic nerve terminals in the proper organization^4,5^. Genetic depletion of the embryonic AChR γ-subunit in muscle fibers markedly decreases staining for AChR clusters with progressive accumulation of synaptic vesicles in the presynaptic terminal^6^, suggesting that embryonic AChR clustering is required for further pre- and postsynaptic development. AChR clustering and formation of the neuromuscular junction (NMJ) are mediated by SMN-derived and muscle-derived molecules. Neuronal agrin released from the nerve terminal^7^ binds to the low-density lipoprotein receptor-related protein 4 (LRP4) on the motor endplate and promotes phosphorylation of the muscle-specific kinase (MuSK) to induce AChR clustering^8,9^. Other SMN-derived molecules driving AChR clustering and NMJ formation include neuregulin 1 and ACh^2,10–15^. In addition, muscle-derived molecules including laminins, FGFs, collagens, BDNF, GDNF, Wnts, and TGF-β^4^ are implicated in orchestrating pre- and postsynaptic NMJ formation.

Wnts are secreted glycoproteins, and Wnt ligands (Wnt4, Wnt9a, Wnt9b, Wnt10b, Wnt11, and Wnt16) are able to promote AChR clustering independent of agrin^16,17^. Wnt ligands bind to the Frizzled-like domain of MuSK, and specific deletion of this MuSK domain compromises AChR clustering^18^. In addition, β-catenin directly promotes AChR clustering by directly binding to rapsyn and by linking rapsyn with the α-catenin-associated cytoskeleton^19^.

R-spondins (Rspo1-4) play essential roles in development through Wnt signaling pathways in vertebrates^20–22^. As a member of the Rspo family, Rspo2 binds directly to the leucine-rich-repeat-containing G-protein coupled receptors (Lgr) 4 and 5 to enhance stabilization of Wnt receptors on the cell membrane and to activate Wnt signaling pathways^23,24^. Rspo2 is implicated in development of the larynx, lung, limb, and trachea in mice^25,26^. *Rspo2*-deficient mice present with an abnormally short left hindlimb and lack of digits on both forelimbs at embryonic day (E) 18.5, and mice die perinatally due to respiratory distress^25,27–29^. In cultured myogenic C2C12 cells, Rspo2 is able to promote muscle differentiation through Wnt/β-catenin signaling^30^. We recently reported by laser capture microdissection of mouse SMNs that Rspo2 is highly expressed in SMNs^31^. Rspo2 is likely to be excreted from the axon terminals of SMNs and is accumulated at the NMJ in wild-type mice. In cultured C2C12 myotubes, Rspo2 induces MuSK phosphorylation and AChR clustering, which requires Wnt ligands but not agrin. Lgr5 is associated with MuSK at the NMJ and serves as a receptor for Rspo2. In *Rspo2*-knockout mice, the number and density of AChRs at the NMJ are reduced with widened synaptic clefts, sparse synaptic vesicles, and markedly reduced frequency of miniature endplate potentials. We assumed that SMN-derived Rspo2 was anchored at the NMJ and promoted AChR clustering as well as NMJ formation, but Rspo2 is also expressed marginally in the diaphragm at E18.5 and in adults^31^. To dissect the differential roles of SMN-derived Rspo2 and muscle-derived Rspo2 on AChR clustering and NMJ formation in mouse embryos, we generated transgenic mice expressing Rspo2 specifically in SMNs or in skeletal muscle on the background of *Rspo2*^−/−^.

## Results

### Specific expression of Rspo2 in the skeletal muscles and SMNs in Rspo2^−/−^ mice

**Figure 1:**
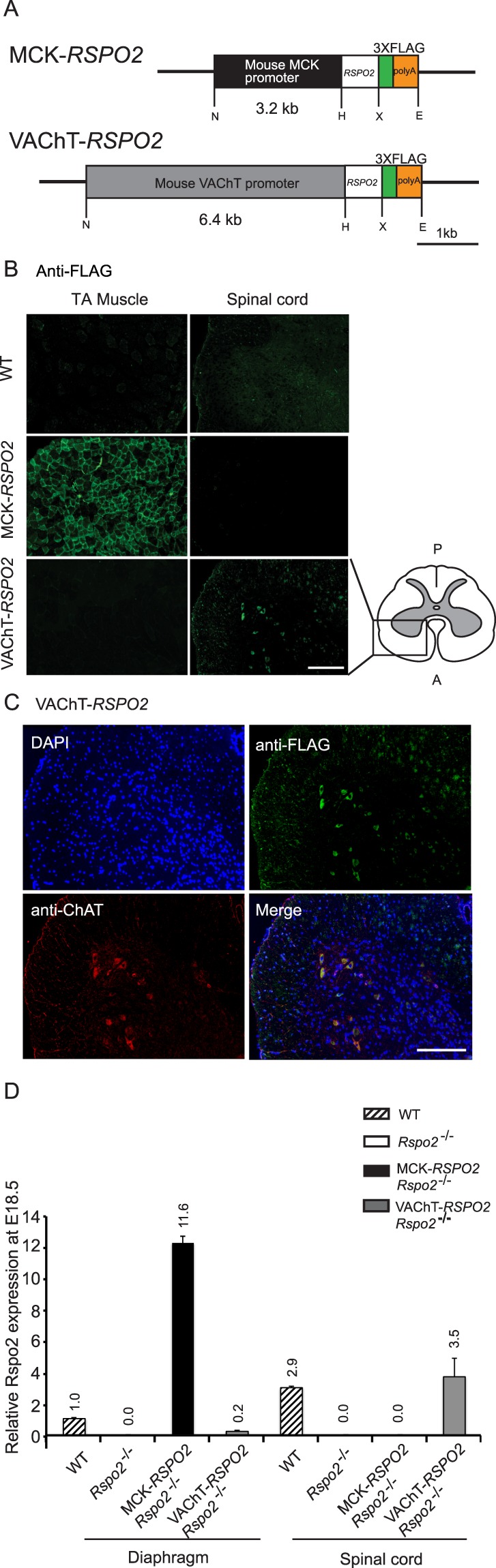
Transgene constructs and *RSPO2* expression in transgenic mice. **(A)** Schematic presentation of transgene constructs for generation of transgenic mice. The muscle-specific MCK promoter and the SMN-specific VAChT promoter were inserted 5′ to the human *RSPO2* gene. Three copies of FLAG cDNA (3 X FLAG) were inserted 3′ to *RSPO2*. N, *Not*I; H, *Hin*dIII; X, *Xba*I; and E, *Eco*RI. **(B)** Immunofluorescent staining of sections of the tibialis anterior (TA) muscle and spinal cord of wild-type (WT) and transgenic adult mice with anti-FLAG antibody. FLAG-tagged Rspo2 was detected in the tibialis anterior of MCK*-RSPO2* mice and in the spinal cord of VAChT*-RSPO2* mice. A, anterior; and P, posterior. Scale bar =100 μm. **(C)** Immunofluorescent staining of the spinal cord of VAChT*-RSPO2* transgenic adult mice with anti-FLAG (green) antibody for detecting Rspo2 and anti-ChAT (red) antibody for visualizing SMNs. FLAG-tagged Rspo2 was primarily expressed in ChAT-positive SMNs, but leaky expression was also observed in a small fraction of cells. Scale bar =100 μm. **(D)** Quantitative RT-PCR of *Rspo2* transcripts. Total RNA was isolated from the diaphragms and spinal cords of wild-type (WT), *Rspo2*^−/−^, MCK*-RSPO2/Rspo2*^−/−^, and VAChT-*RSPO2/Rspo2*^−/−^ embryos at E18.5. Values are normalized to *Gapdh*. The normalized *Rspo2* expression levels are plotted relative to that in the WT diaphragm to make direct comparisons between the diaphragm and spinal cord possible. Mean and SEM (*n* = 3) are indicated.

To understand the origin of Rspo2 that promotes AChR clustering at the NMJ, transgenic mice expressing *Rspo2* specifically in skeletal muscle or SMNs were generated according to the conventional transgenic mice strategy^32^. Human *RSPO2* cDNA with three repeats of FLAG tags at the 3′ end was inserted downstream of either a 3.2-kb mouse muscle creatine kinase (MCK) promoter or a 6.4-kb mouse vesicular acetylcholine transporter (VAChT) promoter (Fig. 1A). The constructs were microinjected into fertilized eggs. PCR genotyping of embryos and adults was carried out with primers targeted to the injected DNA sequence (Supplementary Table S1). Transgenic mice carrying MCK*-RSPO2* and VAChT*-RSPO2* were morphologically normal, viable, active, and fertile without shortened life spans. To confirm expression of FLAG-tagged Rspo2 protein, frozen sections of the tibialis anterior (TA) muscle and the spinal cord from MCK*-RSPO2* and VAChT*-RSPO2* mice were stained with anti-FLAG antibody. MCK*-RSPO2* mice showed the FLAG signal predominantly in the periphery of muscle fibers of TA but not in the spinal cord (Fig. 1B). In contrast, VAChT*-RSPO2* mice showed the FLAG signal in large cells in the ventral horn area of the spinal cord but not in TA (Fig. 1B). Double immunostaining of the spinal cord of VAChT-*RSPO2* mice for the FLAG signal and choline acetyltransferase (ChAT), a marker for motor neurons, revealed that most large cells were double-positive for FLAG and ChAT; however, some FLAG-positive cells were not strongly labeled with ChAT, indicating leaky expression of FLAG-*RSPO2* in non-motor neurons (Fig. 1C). Thus, Rspo2 was specifically expressed in the skeletal muscle of MCK*-RSPO2* mice and in SMNs of VAChT*-RSPO2* mice.

To produce MCK*-RSPO2*/*Rspo2*^+/−^ and VAChT*-RSPO2*/*Rspo2*^+/−^ mice, F1 generations were crossed with heterozygous *Rspo2* knockout (*Rspo2*^+/−^) mice. The resulting MCK*-RSPO2*/*Rspo2*^+/−^ and VAChT*-RSPO2*/*Rspo2*^+/−^ mice were morphologically normal, viable, active, and fertile without shortened life spans. Finally, the heterozygous littermates were intercrossed to generate MCK*-RSPO2/Rspo2*^−/−^ and VAChT*-RSPO2/Rspo2*^−/−^ embryos. Quantitative RT-PCR showed that expression levels of human *RSPO2* in the diaphragm of MCK*-RSPO2*/*Rspo2*^−/−^ mice at E18.5 and in the spinal cord of VAChT*-RSPO2*/*Rspo2*^−/−^ mice at E18.5 were 11.6-fold and 1.23-fold higher, respectively, than the expression levels of *Rspo2* in wild-type mouse embryos at E18.5 (Fig. 1D). The difference between 11.6- and 1.23-folds may represent the difference between the promoter activities of MCK and VAChT.

### Embryonic lethality and morphological defects in the left hindlimb and digits of both forelimbs are not rescued by Rspo2 expression in skeletal muscles or SMNs

**Figure 2:**
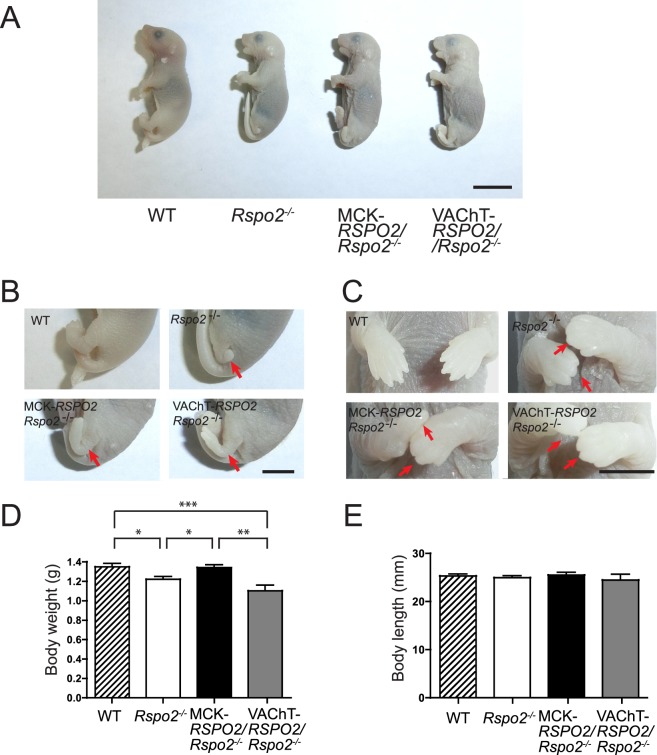
Gross appearance of wild-type (WT), *Rspo2*^−/−^, MCK*-RSPO2/Rspo2*^−/−^, and VAChT*-RSPO2/Rspo2*^−/−^ embryos at E18.5. **(A)** Gross appearance of embryos at E18.5. Scale bar = 10 mm. **(B)** Defect of the left hindlimb (red arrow) in *Rspo2*^−/−^, MCK*-RSPO2/Rspo2*^−/−^, and VAChT*-RSPO2/Rspo2*^−/−^ embryos at E18.5. In VAChT*-RSPO2/Rspo2*^−/−^ embryo, the left hindlimb was extended with a large deformity. Scale bar =5 mm. **(C)** The distal forelimbs at E18.5. Arrows point to defective claws in *Rspo2*^−/−^, MCK*-RSPO2/Rspo2*^−/−^, and VAChT*-RSPO2/Rspo2*^−/−^ mice. Scale bar = 5 mm. **(D**,**E)** The body weight and length were quantified at E18.5. Mean ± SEM (*n* = 10) are indicated. *p*-value = 0.0002 by one way ANOVA for **(D)**. *p < 0.05, **p < 0.01, ***p < 0.001 by post-hoc Tukey test.

Rspo2 is required for normal development of the lungs and limbs^25^. In accordance with a previous report^29^, *Rspo2*^−/−^ mice had abnormally short left hindlimbs and lacked digits of both forelimbs at E18.5 (Fig. 2A–C), and they died shortly after birth due to respiratory distress. MCK*-RSPO2/Rspo2*^−/−^ and VAChT*-RSPO2/Rspo2*^−/−^ mice also died perinatally. Similarly, abnormal morphology of the left hindlimb and missing digits in forelimbs (Fig. 2B,C) were also observed in both MCK*-RSPO2/Rspo2*^−/−^ and VAChT*-RSPO2/Rspo2*^−/−^ embryos at E18.5. The body weights of *Rspo2*^−/−^ and VAChT*-RSPO2/Rspo2*^−/−^ mice at E18.5 were 15% or more lower than those of wild-type mice (Fig. 2D). However, the body weights of MCK*-RSPO2/Rspo2*^−/−^ mice at E18.5 were similar to those of wild-type mice. In contrast, the body lengths of wild-type, *Rspo2*^−/−^, MCK*-RSPO2/Rspo2*^−/−^, and VAChT*-RSPO2/Rspo2*^−/−^ embryos were similar to each other (Fig. 2E).

### SMN-derived Rspo2 regulates the bandwidth of AChR clusters and axonal branching in the diaphragm

**Figure 3:**
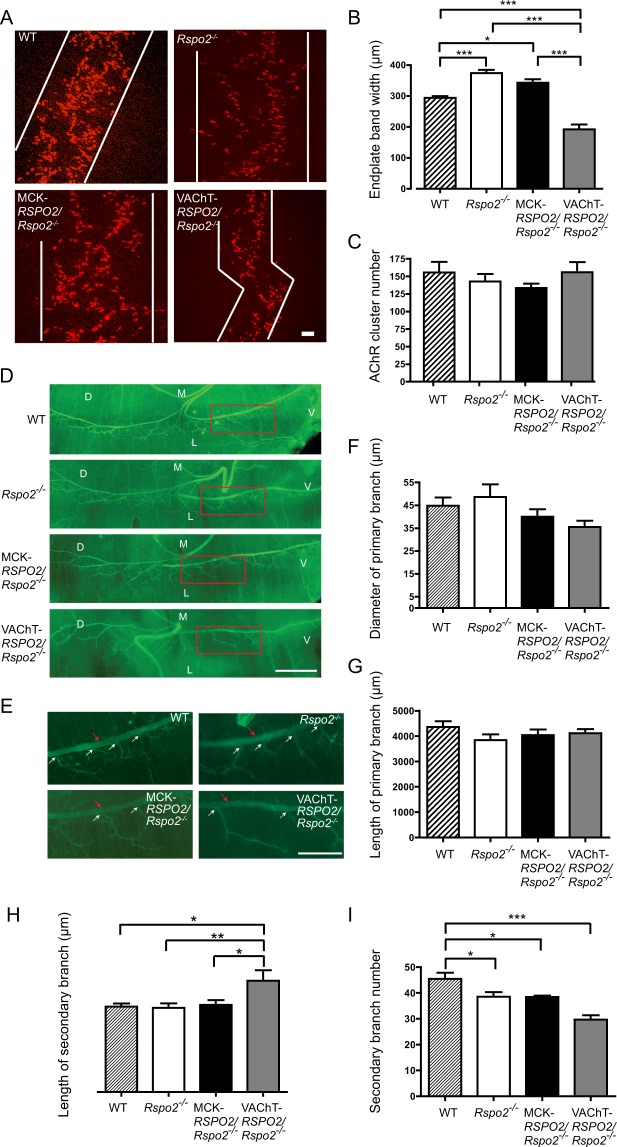
AChR clusters and nerve branches in the diaphragms at low-magnification in wild-type (WT), *Rspo2*^−/−^, MCK*-RSPO2/Rspo2*^−/−^, and VAChT*-RSPO2/Rspo2*^−/−^ embryos at E18.5. **(A)** Representative images of the whole-mount left hemi-diaphragms at E18.5 stained with α-bungarotoxin (red). White lines delineate endplate bands. Scale bar = 50 μm. **(B**,**C)** Blinded morphometric analyses of the endplate bandwidth **(B)** and the number of AChR clusters **(C)** at E18.5. Mean ± SEM (*n* = 5) are indicated. *p*-value < 0.0001 by one way ANOVA. *p < 0.05, **p < 0.01, ***p < 0.0001 by post-hoc Tukey test. **(D)** Representative images of the whole-mount left hemi-diaphragms at E18.5 stained with anti-peripherin antibody (green). D, dorsal; V, ventral; L, lateral; and M, medial. Scale bar = 500 μm. **(E)** Enlarged images of the ventral regions (red squares) in **(D)**. Red arrows point to the primary branches. The primary branches are comprised of two axonal branches originating from the phrenic nerve and extending to the ventral and dorsal sides. White arrows point to representative branch points of the secondary branches. The secondary branches originate from one of the primary branches and end at the NMJ (see Supplementary Fig. S1A for entire representative stretches of the secondary branches). The third branches frequently start from the middle of the secondary branch, and we assumed that the longest axonal branch constituted the secondary branch. Scale bar = 250 μm. **(F–I)** Blinded morphometric analyses of the diameters of primary branches **(F)**, the lengths of primary branches **(G)**, the lengths of secondary branches **(H)**, and the number of secondary branches **(I)**. We counted the total number of secondary branches in the whole mount staining of the left diaphragm. Note that panels **(F–I)** indicate absolute values to compare WT, *Rspo2*^−/−^, MCK*-RSPO2/Rspo2*^−/−^, and VAChT*-RSPO2/Rspo2*^−/−^ embryos. Mean ± SEM (*n* = 5) are indicated. *p*-value = 0.008 **(H)** and *p*-value < 0.0001 **(I)** by one way ANOVA. *p < 0.05, **p < 0.01, ***p < 0.001 by post-hoc Tukey test.

We next examined AChR clustering at the NMJs of the left hemi-diaphragms at low-magnification in wild-type, *Rspo2*^−/−^, MCK*-RSPO2/Rspo2*^−/−^, and VAChT*-RSPO2/Rspo2*^−/−^ mice at E18.5. As all mice died shortly after birth, and as immaturity of the NMJs in mouse embryos made it difficult to obtain dependable results, we analyzed the NMJs only at E18.5. First, we observed that innervation patterns were different in these mice. As we previously reported^31^, *Rspo2*^−/−^ mice formed broader bands of AChR clusters compared to those in wild-type mice. MCK*-RSPO2/Rspo2*^−/−^ mice exhibited similarly broad bands of AChR clusters, whereas VAChT*-RSPO2/Rspo2*^−/−^ mice showed markedly narrow bands of AChR clusters (Fig. 3A,B). In contrast, the numbers of AChR were not changed in wild-type, *Rspo2*^−/−^, MCK*-RSPO2/Rspo2*^−/−^, and VAChT*-RSPO2/Rspo2*^−/−^ mice at E18.5 (Fig. 3C). These findings indicate that SMN-derived Rspo2 ensures proper distribution of AChR clusters.

The axonal branches in the left hemi-diaphragms were observed by immunostaining with anti-peripherin antibody (Fig. 3D,E). The diameters and lengths of the first branches (red arrows in Fig. 3E) were similar in wild-type, *Rspo2*^−/−^, MCK*-RSPO2/Rspo2*^−/−^, and VAChT*-RSPO2/Rspo2*^−/−^ mice (Fig. 3F,G). In *Rspo2*^−/−^ and MCK*-RSPO2/Rspo2*^−/−^ mice, the lengths of secondary branches (white arrows in Fig. 3E) were similar to those in wild-type mice, but the numbers of secondary branches were decreased (Fig. 3G,H). VAChT*-RSPO2/Rspo2*^−/−^ mice showed more prominent changes in secondary branches; the lengths of secondary branches were increased (Fig. 3G) and their numbers were decreased more than those in *Rspo2*^−/−^ mice (Fig. 3H). We also observed that almost all AChR clusters were similarly innervated by motor axons in wild-type, *Rspo2*^−/−^, MCK*-RSPO2/Rspo2*^−/−^, and VAChT*-RSPO2/Rspo2*^−/−^ mice (Supplementary Fig. S1A,B). This suggests that the lack of Rspo2 or overexpression of Rspo2 is unlikely to have a substantial effect on the gross innervation of AChR clusters. Thus, a comparison of *Rspo2*^−/−^ mice against MCK*-RSPO2/Rspo2*^−/−^ and VAChT*-RSPO2/Rspo2*^−/−^ mice revealed that SMN-derived Rspo2 contributes to branching and outgrowth of SMN axons, whereas muscle-derived Rspo2 has no substantial effect on SMN axons.

### SMN-derived Rspo2 regulates the sizes of AChR clusters and nerve terminals

**Figure 4:**
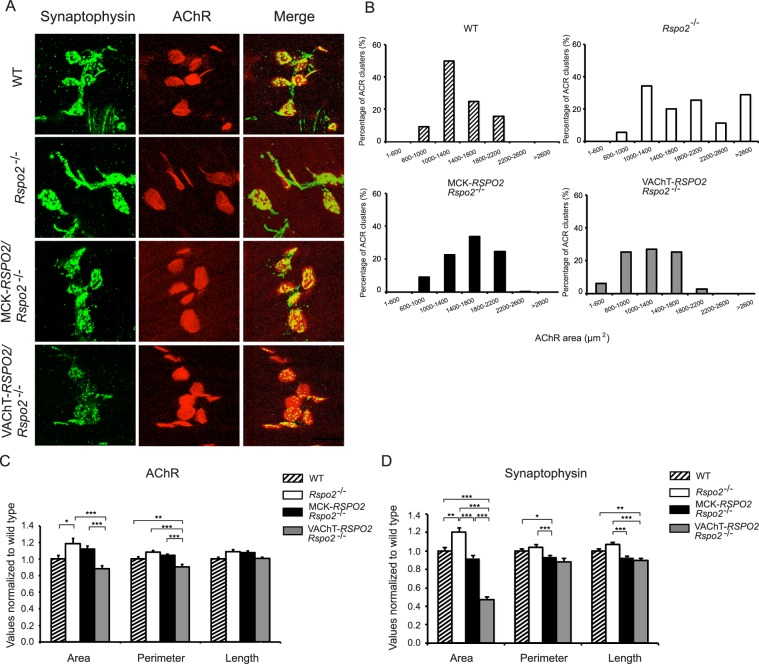
AChR clusters and nerve terminals in the diaphragms at high-magnification in wild-type (WT), *Rspo2*^−/−^, MCK*-RSPO2/Rspo2*^−/−^, and VAChT*-RSPO2/Rspo2*^−/−^ embryos at E18.5. **(A)** Representative confocal images of the NMJs of the right hemi-diaphragms at E18.5 stained with anti-synaptophysin antibody (green) and α-bungarotoxin (red) to visualize the nerve terminals and acetylcholine receptors (AChRs), respectively. Scale bar = 25 μm. **(B)** Size distributions of AChR clusters (*n* = 32 NMJs for each group) by morphometric analysis. AChR cluster sizes were shifted to the right and were broadly distributed in *Rspo2*^−/−^ mice. MCK*-RSPO2/Rspo2*^−/−^ narrowed the distribution, but had no effect on the average size [see **(C)**]. In contrast, VAChT*-RSPO2/Rspo2*^−/−^ rescued the right shift. Statistical differences of AChR areas by one-way ANOVA are indicated in **(C)**. **(C**,**D)** Morphometric analyses of the area, perimeter, and length of α-bungarotoxin-positive signals for AChR clusters **(C)** and synaptophysin-positive signals for nerve terminals **(D)**. **(B**–**D)** AChR areas and synaptophysin-positive areas were manually traced individually and measured by MetaMorph software. The perimeter is the circumference of the traced area. The length is defined as the longest axes of the traced area. Mean ± SEM (*n* = 32 NMJs) are indicated. When the *p*-value by one-way ANOVA is less than 0.05, *p*-values by post-hoc Tukey test are indicated by *p < 0.05, **p < 0.01 and ***p < 0.001.

We next investigated AChR clustering at the NMJs of the right hemi-diaphragms at high-magnification in wild-type, *Rspo2*^−/−^, MCK*-RSPO2/Rspo2*^−/−^, and VAChT*-RSPO2/Rspo2*^−/−^ mice at E18.5. AChRs and nerve terminals were stained by α-bungarotoxin and anti-synaptophysin antibody, respectively (Fig. 4A). The distribution of AChR cluster areas was shifted toward larger sizes in *Rspo2*^−/−^ mice (Fig. 4B), as we previously reported^31^. SMN-derived Rspo2 in VAChT*-RSPO2/Rspo2*^−/−^ mice corrected the abnormally enlarged AChR cluster areas (Fig. 4B,C) and over-corrected abnormally enlarged presynaptic synaptophysin-positive areas (Fig. 4D). Muscle-derived Rspo2 in MCK*-RSPO2/Rspo2*^−/−^ mice had no effect on AChR clusters (Fig. 4B,C), but normalized synaptophysin-positive areas (Fig. 4D). These data support the aforementioned observation at low-magnification that SMN-derived Rspo2 is likely to have more of an effect on AChR clustering and synaptophysin-positive nerve terminals than muscle-derived Rspo2.

### Muscle-derived Rspo2 regulates juxtaposition of the active zones and synaptic folds

**Figure 5:**
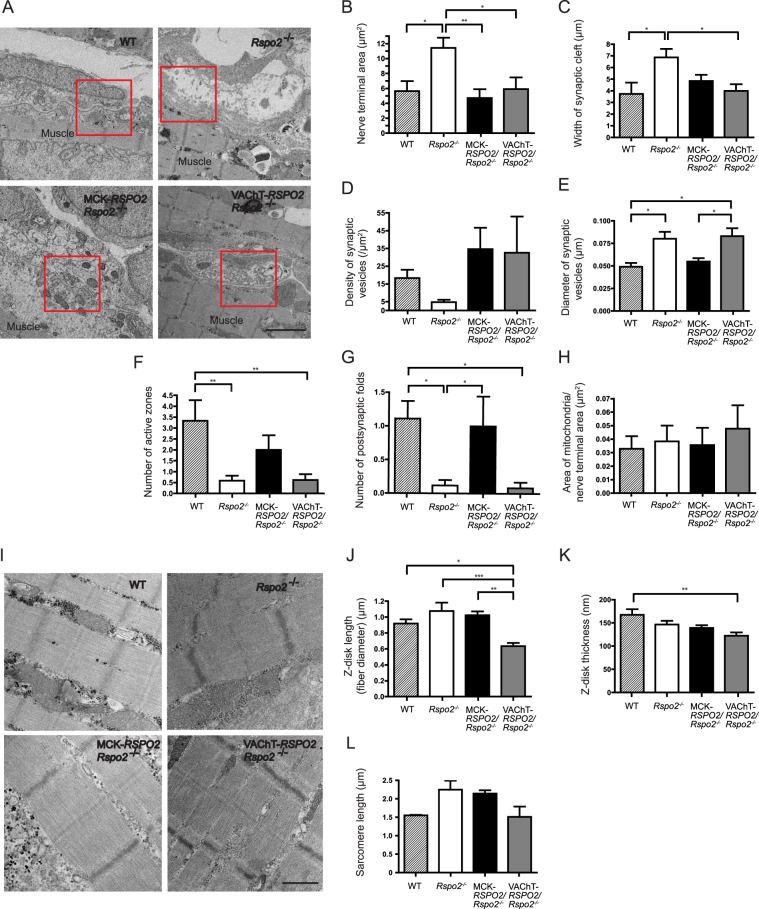
Ultrastructures of the NMJ and the tibialis anterior in wild-type (WT), *Rspo2*^−/−^, MCK*-RSPO2/Rspo2*^−/−^, and VAChT*-RSPO2/Rspo2*^−/−^ embryos at E18.5. **(A)** Representative electron micrographs of the NMJs in the left diaphragms at E18.5. Scale bar = 2 μm. **(B–H)** Blinded morphometric analyses of the nerve terminal area **(B)**, the widths of synaptic clefts **(C)**, the density of synaptic vesicles **(D)**, the diameters of synaptic vesicles **(E)**, the number of active zones per nerve terminal **(F)**, the number of postsynaptic folds **(G)**, and the area of mitochondria normalized to the nerve terminal area **(H)**. Mean ± SEM (*n* = 10 NMJs) are indicated. When the *p*-value by one-way ANOVA is less than 0.05, *p*-values by post-hoc Tukey test are indicated by *p < 0.05, **p < 0.01, and ***p < 0.001. SV, synaptic vesicles. **(I)** Representative electron micrographs of the left diaphragm muscles at E18.5. Scale bar = 1 μm. **(J–L)** Blinded morphometric analyses of the length **(J)** and thickness **(K)** of Z-disk, as well as the sarcomere length **(L)**. Mean ± SEM (*n* = 10) are indicated. *P*-value = 0.003 **(J)** and *p*-value = 0.001 **(K)** by one way ANOVA. *p < 0.05, **p < 0.01, ***p < 0.001 by post-hoc Tukey test.

The NMJs at E18.5 were ultrastructurally visualized (Fig. 5A) and were morphometrically analyzed in a blinded manner (Fig. 5B–H). Compared to wild-type mice, the areas of nerve terminals (Fig. 5B) and widths of synaptic clefts (Fig. 5C) were increased in *Rspo2*^−/−^ mice, and both were normalized in MCK*-RSPO2/Rspo*2^−/−^ and VAChT*-RSPO2/Rspo2*^−/−^ mice. Similarly, the densities of synaptic vesicles were decreased in *Rspo2*^−/−^ mice and were over-corrected in both MCK*-RSPO2/Rspo*2^−/−^ and VAChT*-RSPO2/Rspo2*^−/−^ mice (Fig. 5D). In contrast, the diameters of synaptic vesicles were increased in *Rspo2*^−/−^ mice, and they were normalized in MCK*-RSPO2/Rspo*2^−/−^ mice, but not in VAChT*-RSPO2/Rspo*2^−/−^ mice (Fig. 5E). Similarly, the numbers of active zones (Fig. 5F) and the numbers of postsynaptic folds (Fig. 5G) were markedly decreased in *Rspo2*^−/−^ mice, and they were partially normalized in MCK*-RSPO2/Rspo*2^−/−^ mice but not in VAChT*-RSPO2/Rspo*2^−/−^ mice. In contrast to the above parameters, the areas of mitochondria at the nerve terminals were similar in wild-type, *Rspo*2^−/−^, MCK*-RSPO2/Rspo*2^−/−^, and VAChT*-RSPO2/Rspo2*^−/−^ mice, indicating that Rspo2 plays no role in the development of synaptic mitochondria (Fig. 5H). To summarize, muscle-derived Rspo2 (MCK*-RSPO2*) normalized all six abnormal parameters observed in *Rspo2*^−/−^ mice (Fig. 5B–G), whereas SMN-derived Rspo2 (VAChT*-RSPO2*) was able to normalize only three of them (Fig. 5B–D).

### SMN-derived Rspo2 regulates muscle differentiation

Ultrastructural analysis of the diaphragm revealed that the Z-disk length (Fig. 5J), representing the myofibril diameter and the sarcomere length (Fig. 5L) tended to be increased in *Rspo2*^−/−^ mice, which were corrected or over-corrected in VAChT*-RSPO2/Rspo2*^−/−^ mice, but not in MCK*-RSPO2/Rspo2*^−/−^ mice. In contrast, the Z-disk thickness tended to be decreased in *Rspo2*^−/−^ mice, which was not mitigated in VAChT*-RSPO2/Rspo2*^−/−^ or MCK*-RSPO2/Rspo2*^−/−^ mice (Fig. 5K). Thus, SMN-derived Rspo2 is likely to regulate muscle differentiation, which may be secondary to the ameliorated AChR clustering observed with SMN-derived Rspo2.

### SMN-derived Rspo2 nearly normalizes abnormally regulated genes in *Rspo2*^−/−^ mice

**Figure 6:**
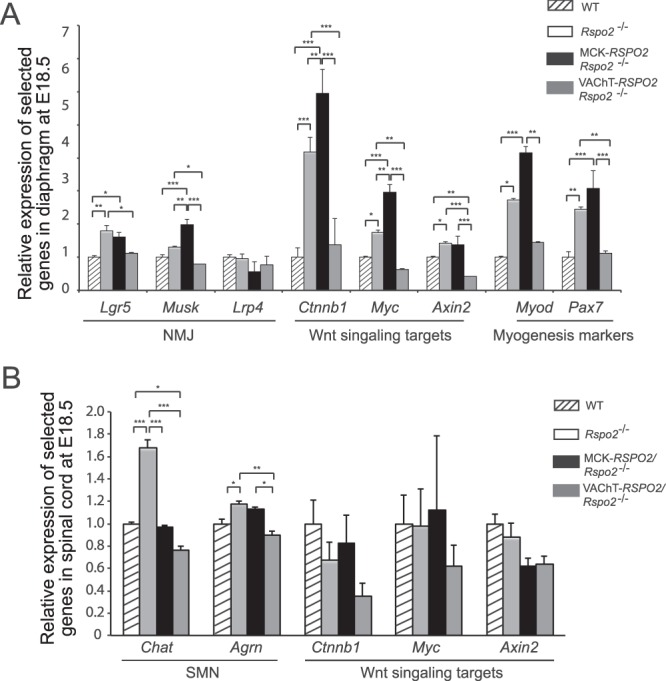
Quantitative RT-PCR (qRT-PCR) analysis of the diaphragms and spinal cords in wild-type (WT), *Rspo2*^−/−^, MCK*-RSPO2/Rspo2*^−/−^, and VAChT*-RSPO2/Rspo2*^−/−^ embryos at E18.5. **(A)** qRT-PCR of genes for the NMJ (*Lgr5*, *MuSK*, and *Lrp4*), Wnt signaling targets (*Ctnnb1*, *Myc*, and *Axin2*), and myogenesis markers (*Myod* and *Pax7*) in the diaphragms. **(B)** qRT-PCR of genes for the SMNs (*Chat* and *Agrn*) and Wnt signaling targets (*Ctnnb1*, *Myc*, and *Axin2*) in the spinal cords. **(A**,**B)** mRNA levels were normalized for beta2-microglobulin (*B2m*) and also for wild-type mice. Mean ± SEM (*n* = 3 mice) are indicated. When the *p*-value by one-way ANOVA is less than 0.05, *p*-values by post-hoc Tukey test are indicated by *p < 0.05, **p < 0.01, and ***p < 0.001.

To differentiate the effects of muscle-derived Rspo2 and SMN-derived Rspo2 on the NMJ, we examined gene expression levels in the diaphragm and spinal cords of wild-type, *Rspo2*^−/−^, MCK*-RSPO2/Rspo2*^−/−^, and VAChT*-RSPO2/Rspo2*^−/−^ mice at E18.5. In Wnt/β-catenin signaling, Wnt protein binds to its receptors (Frizzled and LRP5/6) to inhibit degradation of the co-transcriptional factor β-catenin, encoded by *Ctnnb1*, which upregulates *Myc* encoding c-myc, *Axin2* encoding Axin2, and others^33–37^. The target genes of Wnt/β-catenin signaling (*Ctnnb1*, *Myc*, and *Axin2*) were increased in the diaphragms of *Rspo2*^−/−^ mice (Fig. 6A). Muscle-derived Rspo2 failed to rescue the abnormal increases, but SMN-derived Rspo2 normalized them. In contrast, these genes were not changed in the spinal cords of *Rspo2*^−/−^ mice, and neither muscle-derived Rspo2 nor SMN-derived Rspo2 had any additional effect (Fig. 6B).

We previously reported that Rspo2 enhances MuSK phosphorylation by binding to the Lgr5 receptor expressed at the NMJ^31^. In accordance with our previous report, gene expression levels of *Lgr5* and *MuSK* were increased in *Rspo2*^−/−^ diaphragms (Fig. 6A). Muscle-derived Rspo2 failed to normalize the increases, but SMN-derived Rspo2 normalized them. These results suggest that SMN-derived Rspo2 plays a pivotal role in regulation of the Wnt/β-catenin signaling and subsequent AChR clustering at the NMJ.

The expression levels of myogenesis marker genes of *Myod* and *Pax7* were increased in *Rspo2*^−/−^ diaphragms (Fig. 6A). Muscle-derived Rspo2 further enhanced the increases, whereas SMN-derived Rspo2 normalized them. Ultrastructurally, the aforementioned increased myofibril sizes in *Rspo2*^−*/*−^ and MCK*-RSPO2/Rspo*2^−/−^ mice (Fig. 5J) may be partly accounted for by increased expression levels of myogenesis marker genes. Macroscopically, however, gross measurement of the diaphragm sizes revealed that the diaphragm thickness tended to be rather decreased in *Rspo2*^−/−^ and MCK*-RSPO2/Rspo*2^−/−^ mice (Supplementary Fig. S1C–G). The increased *Myod* and *Pax7* in these mice were likely a compensation for immature myogenesis due to defective NMJ signal transmission as observed in patients with congenital myasthenic syndromes^38,39^, but the compensation was likely to be macroscopically insufficient.

The expression of SMN-specific *Chat* was markedly elevated in the spinal cords of *Rspo*2^−/−^ mice (Fig. 6B). Both muscle-derived Rspo2 and SMN-derived Rspo2 normalized it. The expression of another SMN-specific gene, *Agrn*, was upregulated in *Rspo*2^−/−^ mice (Fig. 6B). Muscle-derived Rspo2 failed to suppress the increase, whereas SMN-derived Rspo2 normalized it.

To summarize, seven out of eight genes (*Lgr5*, *MuSK*, *Ctnnb1*, *Myc*, *Axin2*, *Myod*, and *Pax7*) were abnormally increased in *Rspo2*^−/−^ diaphragms (Fig. 6A). Muscle-derived Rspo2 failed to normalize the increases, whereas SMN-derived Rspo2 normalized them. In contrast, two out of five genes (*Chat* and *Agrn*) were increased in the spinal cord of *Rspo*2^−/−^ mice (Fig. 6B). SMN-derived Rspo2 normalized both genes, whereas muscle-derived Rspo2 only normalized *Chat*.

## Discussion

We rescued the phenotypes of *Rspo2*^−/−^ mice by MCK-promoter-driven muscle-specific Rspo2 and VAChT-promoter-driven SMN-specific Rspo2. Rspo2 plays critical roles in the development of multiple tissues including the craniofacial structures, kidneys, lungs, and limbs in mice and humans^25,28,40^. *Rspo2*^−/−^ mice die shortly after birth due to respiratory distress, and they have defects of the left hindlimb and digits of both forearms^25,29^. Neither muscle-derived Rspo2 nor SMN-derived Rspo2 rescued the perinatal death or limb anomalies (Fig. 2A–C). Rspo2 promotes myogenic differentiation and hypertrophic myofiber formation in C2C12 myoblasts^20,22,41^. Normalization of the reduced body weight in *Rspo2*^−/−^ mice by muscle-derived Rspo2 (Fig. 2D) may be partly accounted for by the effect of Rspo2 on myogenesis. Especially, non-physiological overexpression of Rspo2 by the MCK promoter in MCK-*RSPO2/Rspo2*^−/−^ mice (Fig. 1D) might have helped normalize the body weight. In contrast, SMN-derived Rspo2 had no effect on the reduced body weight (Fig. 2D). SMN-derived Rspo2 reduced the abnormally increased muscle fiber sizes in *Rspo2*^−/−^ mice (Fig. 5J,L), which might have cancelled the effect of Rspo2 on muscle mass. As Rspo2 is a secreted protein, both SMN-derived Rpos2 and muscle-derived Rspo2 can reach the target tissues and exert overlapping effects. SMN-specific and muscle-specific knockdown of *Rspo2* would have similar overlapping phenotypes. Identifying the origin of a secreted protein is thus challenging and enigmatic.

**Figure 7:**
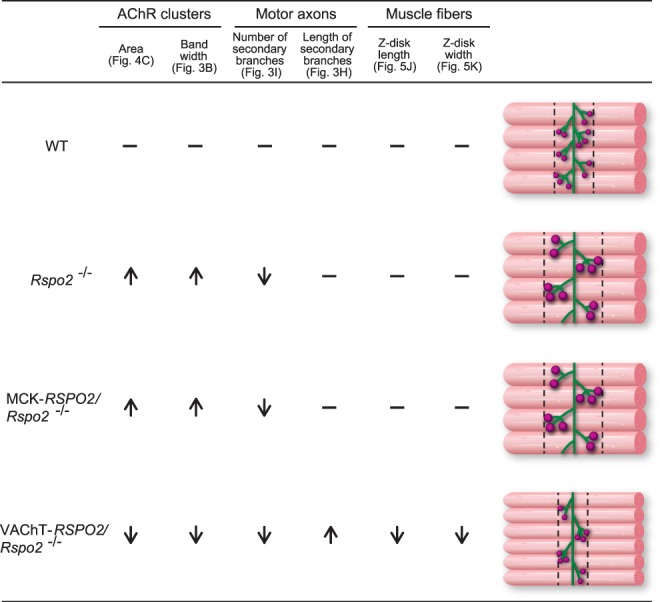
Schematic summary of NMJs and skeletal muscles in wild-type (WT), *Rspo2*^−/−^, MCK*-RSPO2/Rspo2*^−/−^, and VAChT*-RSPO2/Rspo2*^−/−^ embryos at E18.5. Arrows indicate increased or decreased as compared to WT embryos. Hyphens indicate similar to wild-type embryos. Schematic diagrams of nerve branches (green) and AChR clusters (red) are shown on the right side. Endplate bands are delimited by dotted lines.

**Figure 8:**
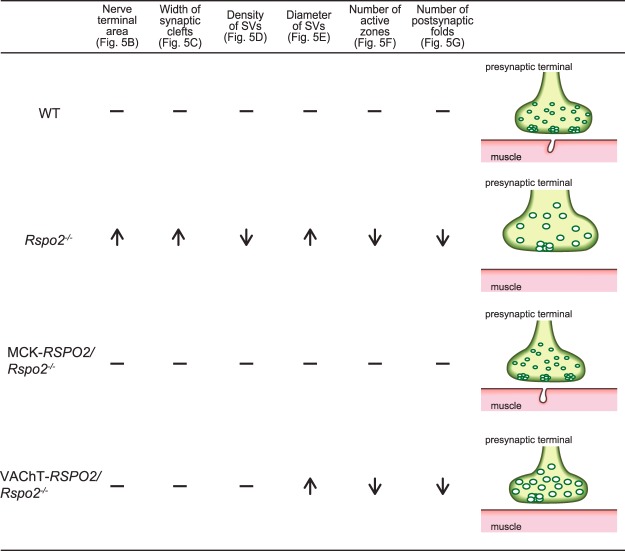
Schematic summary of NMJ ultrastructures in wild-type (WT), *Rspo2*^−/−^, MCK*-RSPO2/Rspo2*^−/−^, and VAChT*-RSPO2/Rspo2*^−/−^ embryos at E18.5. Arrows indicate increased or decreased as compared to WT embryos. Hyphens indicate similar to wild-type embryos. Schematic diagrams of presynaptic nerve terminals with synaptic vesicles (green) and postsynaptic folds (red) are shown on the right side.

The methods of our NMJ analyses can be divided into four categories: (i) low-magnification analysis of the whole-mount diaphragms (Figs 3 and 7), (ii) high-magnification analysis of the NMJ (Figs 4 and 7), (iii) ultrastructural analysis of the NMJ (Figs 5 and 8), and (iv) gene expression analysis of the diaphragm and spinal cord (Fig. 6). In three of the four categories (i, ii, and iv), SMN-derived Rspo2 mitigated aberrant NMJ features in *Rspo2*^−/−^ mice more than muscle-derived Rspo2. Ultrastructural features (iii) were normalized by both SMN-derived and muscle-derived Rspo2, but muscle-derived Rspo2 improved more features than SMN-derived Rspo2. Individual NMJ features can also be divided into three categories by the effects of muscle- and SMN-derived Rspo2: (a) features improved by both muscle-derived and SMN-derived Rspo2, (b) features improved by muscle-derived Rspo2 more than SMN-derived Rspo2, and (c) features improved by SMN-derived Rspo2 more than muscle-derived Rspo2. First, features improved by both muscle-derived and SMN-derived Rspo2 were three of the six ultrastructural parameters (Figs 5B–D and 8). Elevated expression of the *Chat* gene in the spinal cord was also mitigated by both Rspo2’s (Fig. 6B). Second, features improved by muscle-derived Rspo2 were the other three of the six ultrastructural parameters (Figs 5E,G and 8)). Third, features improved by SMN-derived Rspo2 included the bandwidth of AChR clusters (Figs 3A,B and 7) and the area of AChR clusters (Figs 4C and 7). In the diaphragm, elevated expression levels of NMJ-specific genes, target genes of Wnt/β-catenin signaling, and myogenic marker genes were normalized only by SMN-derived Rspo2 (Fig. 6A). In the spinal cord, elevated expression of *Agrn* was normalized only by SMN-derived Rspo2 (Fig. 6B). Thus, SMN-derived Rspo2 improved the NMJ phenotypes more than muscle-derived Rspo2. Higher expression of Rspo2 in the spinal cord compared to the diaphragm at E18.5 in wild-type mice (Fig. 1D) also supports the notion that SMN-derived Rspo2 plays a pivotal role in NMJ development. Rspo2 is a secreted protein, but the distance that Rspo2 may travel in our body remains unknown. However, we can assume that Rspo2-secreting cells and their proximal cells are exposed to a high concentration of Rspo2. Indeed, only SMN-derived Rspo2 over-corrected the bandwidth of AChR clusters, which was determined by branching of motor axons. The reason why only muscle-derived Rspo2, but not SMN-derived Rspo2, normalized three ultrastructural features remains unknown, but muscle-derived and SMN-derived Rspo2 may have different roles during normal development of the NMJ. Alternatively, as Lgr5 is a receptor for Rspo2, and is highly expressed in motor neurons including SMNs^42^, muscle-derived Rspo2 might have worked on Lgr5 expressed in SMNs. Additionally, as the MCK promoter drove Rspo2 expression in the skeletal muscle 11.6 times more than that in wild-type skeletal muscle (Fig. 1D), the phenotypic rescue observed in MCK-*RSPO2/Rspo2*^−/−^ mice might be partly accounted for by over secretion of Rspo2 from the skeletal muscle.

Individual NMJ features that were mitigated by either muscle-derived or SMN-derived Rspo2 are further addressed in detail. First, *Rspo2*^−/−^ mice showed broadening of the bandwidth of AChR clusters in the diaphragm. Muscle-derived Rspo2 had no effect on this broadening, but SMN-derived Rspo2 made the bandwidth of AChR clusters even narrower than that in wild-type mice. As the number of AChR clusters remained unchanged (Fig. 3C), SMN-derived Rspo2 was likely to have compacted bands of AChR clusters. Since the VAChT promoter drove the expression of Rspo2 1.23-fold higher than wild-type mice in the spinal cord at E18.5 (Fig. 1D), the phenotype in *Rspo2*^−/−^ mice was likely to be over-corrected by SMN-derived Rspo2. Broadening of the bandwidth of AChR clusters is observed in the mice knocked out for *Agrn*^43,44^, a gene for a core component of the planar cell polarity (PCP) pathway, *Vangl2*^17,44,45^, *Wnt4*^44,46^, *Wnt11*^17,44,45^, and muscle-specific *Ctnnb1* encoding β-catenin^17,44,45^. Similar broadening is observed in mice with muscle-specific overexpression of *Ctnnb1*^17,44,45^, *in utero* co-injection of Wnt4 and Wnt11^17,44,45^, and *in utero* injection of a secreted Wnt antagonist, Dkk1^17,44,45^. In contrast, *in utero* injection of a secreted Wnt antagonist, Sfrp4, narrowed the bandwidth of AChR clusters^17,44,45^. As both injection and knockdown of Wnt4 and Wnt11 give rise to similar broadening of AChR clusters, and as injection of Wnt-antagonizing Dkk1 and Sfrp4 have the opposite effects, finely orchestrated Wnt signaling pathways are likely to be critical for normal alignment of AChR clusters. Second, gene expression of *Agrn* was increased in the spinal cord in *Rspo2*^−/−^ mice. Muscle-derived Rspo2 failed to suppress this increase, whereas SMN-derived Rspo2 normalized *Agrn* expression (Fig. 6B). During the NMJ formation, agrin is also synthesized and released from SMNs to the synaptic clefts and binds to the LRP4/MuSK complex to induce AChR clustering^8,9,47^. Although Rspo2 does not require agrin to induce AChR clustering in myotubes^31^, Rspo2 and agrin released from SMNs are likely to cooperate to form AChR clusters *in vivo*. It is likely that *Rspo2*^−/−^ mice overexpress agrin to compensate for the lack of AChR-inducing Rspo2, and SMN-specific rescue of Rspo2 might have made the compensation unnecessary. Third, the reduced number of secondary axonal branches in *Rspo2*^−/−^ mice was not affected by muscle-derived Rspo2 and was further reduced by SMN-derived Rspo2 (Fig. 3I). The reduced numbers of secondary branches are observed in mice deficient for *Agrn*^43,44^, *Wnt11*^17,44,45^, and muscle-specific *Ctnnb1*^17,44,45^, as well as in mice overexpressing muscle-specific β-catenin^17,44,45^ and *in utero* injected with Sfrp4^17,44,45^. Again, as both up- and down-regulation of Wnt signaling molecules reduces the number of secondary branches, finely tuned activations of Wnt signaling pathways are likely to be crucial for normal terminal arborization of SMN axons. Fourth, muscle-derived Rspo2 rescued six abnormal ultrastructural parameters, whereas SMN-derived Rspo2 rescued only three of them (Fig. 8). The roles of muscle-derived molecules on NMJ formation are demonstrated in mice deficient for *Nedd4*^48^, muscle-specific *Ctnnb1*^44^, γ-AChR subunit^6,49^, and LRP4^50,51^, as well as in mice overexpressing muscle-specific *Ctnnb1*^17,44,45^. Rspo2 may be the fifth muscle-derived molecule that regulates formation of the NMJs. To summarize, we demonstrate that SMN-derived Rspo2 is required for AChR clustering at the motor endplate and for the NMJ formation, and muscle-derived Rspo2 also plays a substantial role in ultrastructural formation of the NMJ.

## Materials and Methods

### Transgene constructs

Human *RSPO2* cDNA (clone ID 100062073) was purchased from Open Biosystems. To generate CMV-*RSPO2*, the coding region of *RSPO2* cDNA was amplified from the clone by PCR (see Supplementary Table S1) and was inserted into the *Hin*dIII and *Xba*l sites of the CMV-driven expression vector, p3XFLAG-CMV-14 (Sigma-Aldrich). A 3198-bp promoter region (positions −3349 to +7 of the ATG translational start site) of the mouse *Ckm* gene encoding MCK and a 6398-bp promoter region (positions −6358 to +41 of the ATG translational start site) of the mouse *Slc18a3* gene encoding VAChT were amplified by PCR (Supplementary Table S1). The amplified promoter regions of MCK and VAChT were inserted into the *Not*I and *Hind*III sites of CMV-*RSPO2* to substitute for the CMV promoter and to generate MCK-*RSPO2* and VAChT-*RSPO2*, respectively (Fig. 1). Lack of PCR artifacts were confirmed by sequencing the entire inserts.

### Generation of transgenic mice

All experiments using mice were approved by the Animal Care and Use Committee of the Nagoya University and were performed in accordance with the relevant guidelines. The MCK- and VAChT*-RSPO2* transgenes were excised from the plasmids with *Not*I and *EcoR*V (Fig. 1). Then, the fragments were purified from a 1% agarose gel using the Wizard SV Gel and PCR Clean-Up System (Promega) and diluted to a final concentration of 100 ng/μl in 50 μl of Nuclease-Free water (Promega). The purified DNA was microinjected into fertilized eggs of C57BL/6J mice. The microinjection and subsequent transfer to foster mothers were performed as reported elsewhere^32^. *Rspo2*-knockout mouse on the C57BL/6J background was kindly provided by Drs. Motoko Aoki and Hitoshi Okamoto at Riken, Japan^29^. MCK*-RSPO2/Rspo2*^−/−^ mice and VAChT*-RSPO2/Rspo2*^−/−^ mice were generated by crossing the MCK*-RSPO2* and VAChT*-RSPO2* transgenic mice with heterozygous *Rspo2*^+/−^ mice. The produced heterozygote mice were then intercrossed to generate the homozygotes. The presence of both alleles was identified by PCR genotyping with primers shown in Supplementary Table S1. Genomic DNA was extracted from the mouse tail using 1 mg/ml proteinase K (Sigma-Aldrich) in DirectPCR Lysis Reagent (Mouse Tail) (Viagen). Tails were digested at 55 °C overnight and at 85 °C for 45 min. After centrifugation at 16,000 × *g* for 5 min, DNA in the supernatant was amplified by PCR for genotyping. Embryos were collected by cesarean sections of pregnant mice because *Rspo2*-deficient mice died shortly after birth.

### Immunofluorescence

The tibialis anterior (TA) muscle and spinal cord were isolated from wild-type adult mice (C57BL/6J), and they were immediately frozen in liquid nitrogen and kept at −80 °C overnight. The muscle was embedded in OCT compound, frozen on dry ice, and dissected into 20-μm sections using a cryostat (CM3050S, Leica Biosystems). For Rspo2 staining, sections were incubated with rabbit polyclonal anti-FLAG antibody (1:100, PM020, MBL) at 4 °C overnight and were then incubated with FITC-conjugated goat anti-rabbit IgG secondary antibody (1:100, FI1000, Vector Laboratories) at 4 °C for 2 h in a humidified chamber. For ChAT staining, sections were incubated with goat polyclonal anti-Choline Acetyltransferase antibody (1:100, AB144P, Millipore) at 4 °C overnight and were then incubated with biotinylated anti-goat IgG (1:300, BA-9500, Vector Laboratories) for 1 h at room temperature (26 °C), followed by incubation with Alexa 546-conjugated streptavidin (1:500, S11225, Invitrogen) for 2 h at 4 °C in a humidified chamber. Sections were mounted in the VectaShield mounting medium with DAPI (Vector Laboratories) and visualized using the IX71 microscope (Olympus).

For microscopic examination of the whole-mount diaphragms, embryos were sacrificed on E18.5, and the diaphragms were dissected and fixed in 2% paraformaldehyde (PFA) in 0.1 M phosphate buffer (pH 7.4) at room temperature (26 °C) for 2 h. After the removal of connective tissue, the whole-mount diaphragm was permeabilized with 0.5% Triton X-100 in 0.1 M phosphate buffer (pH 7.4) for 10 min and then incubated in a blocking buffer containing 3% bovine serum albumin (BSA) and 5% normal goat serum (NGS) at 4 °C overnight. After washing with 0.1 M phosphate buffer (pH 7.4) several times, the diaphragms were incubated overnight with anti-peripherin antibody (1:800, AB1530, Millipore) and anti-synaptophysin antibody (1:100, 180130, Thermo Fisher Scientific). After washing the diaphragms, the diaphragms were incubated with α-Bungarotoxin, Alexa Fluor 594 conjugate (1:100, B13423, Invitrogen) at 4 °C overnight. We quantified the whole-mount diaphragms under an Olympus FSX100 fluorescence microscope by two blinded researchers. The NMJ signals were visualized with an A1Rsi microscope (Zeiss). Unsaturated signals were quantified by two blinded researchers using MetaMorph software (Molecular Devices). We defined that the primary branches are two axonal branches originating from the phrenic nerve and extending to the ventral and dorsal sides. The length of the primary branches was defined as the sum of the two primary branches. The secondary branches originate from one of the primary branches and end at the NMJ. The third branches frequently start from the middle of the secondary branch, and we assumed that the longest axonal branch constituted the secondary branch. The length of the secondary branches was defined as the average of all the secondary branches. For quantitative analyses, Alexa Fluor 594 signals for AChR clusters and FITC signals for synaptophysin were manually traced individually. The areas and the perimeters of the traces were automatically measured by MetaMorph software. The lengths of the longest axes of the traces were also measured by MetaMorph.

### Quantitative RT-PCR

The diaphragms and spinal cords were harvested from E18.5 embryos. Total RNA was extracted using TRIzol Reagent (Invitrogen). The extracted RNA was reverse-transcribed using ReverTra Ace (Toyobo) with Oligo(dT)_20_ Primer (Thermo Fisher Scientific). The synthesized cDNA was quantified using with SYBR Premix Ex Taq II (Takara) by real-time PCR on a LightCycler 480 (Roche). The expression level of each gene was normalized for β2-microglobulin (*B2m*) and also for wild-type mice. The primer pairs are listed in Supplementary Table S2.

### Electron microscopy

Ultrastructure of the left diaphragm at E18.5 was analyzed as previously described^31^. Briefly, the diaphragm and thorax of E18.5 embryos were isolated together, and they were fixed in 4% paraformaldehyde for 3 h while applying physiological tension to the diaphragm by the thorax. The middle portion of the diaphragm muscle fibers, where the NMJs were, was isolated and minced into 0.2 to 0.3-mm blocks. The excised blocks were fixed with 2% glutaraldehyde for 2 h, treated with 1% OsO_4_, dehydrated in ethanol, and embedded in Epon 812 (TAAB). As the phrenic nerve could not be traced to its nerve terminal even in wild-type embryos, every second block was stained for cholinesterase using the Ellman method to confirm that the excised blocks indeed included the NMJs. Ultrathin sections were made from blocks that were not stained for cholinesterase. Ultrathin sections (60 to 70 nm) were stained with uranyl acetate and lead citrate. We identified the NMJs by inspecting the entire ultrathin sections using a JEM-1400 transmission electron microscope. The following morphometric parameters were measured according to a previous report^52^: nerve terminal area in μm^2^, synaptic vesicle density in μm^2^ at the nerve terminal area, area of mitochondria/area of nerve terminal (%), the number of active zones, the diameter of synaptic vesicles, and the width of the synaptic cleft. The active zone was defined as the site with a cluster of five or more synaptic vesicles gathered at the presynaptic membrane^53^. The postsynaptic fold was defined as an indentation in the postsynaptic membrane, where the fold depth was more than 70 nm and the width of the fold aperture was less than a half of the fold depth (see white arrowheads in Supplementary Fig. S2). The numbers of active zones and postsynaptic folds were counted for each nerve terminal. Images were quantified using the ImageJ program (http://imagej.nih.gov/ij/).

## Statistical analyses

Data of multiple groups were analyzed by one-way ANOVA and post-hoc Tukey adjustments using Prism 5.0 (GraphPad) software. *P* values of 0.05 or less were considered statistically significant. Each experiment was conducted three or more times, and the numbers of experiments are indicated in the figure legends.

## Electronic supplementary material

Supplementary Information
