# Supplementary material for: Differential effects of spinal motor neuron-derived and skeletal muscle-derived Rspo2 on acetylcholine receptor clustering at the neuromuscular junction
Source: Sci Rep. 2018 Sep 11;8:13577. doi: 10.1038/s41598-018-31949-7 (PMC6133930; doi:10.1038/s41598-018-31949-7)

Differential effects of spinal motor neuron-derived and skeletal muscle-derived Rspo2 on acetylcholine receptor clustering at the neuromuscular junction

Jin Li, Mikako Ito, Bisei Ohkawara, Akio Masuda, Kinji Ohno

Division of Neurogenetics, Center for Neurological Diseases and Cancer, Nagoya University Graduate School of Medicine, Nagoya University Graduate School of Medicine, Nagoya, Japan

Address correspondence to Dr. Kinji Ohno
Division of Neurogenetics, Center for Neurological Diseases and Cancer, Nagoya University Graduate School of Medicine, 65 Tsurumai, Showa-ku, Nagoya 466-8550, Japan.
e-mail: ohnok@med.nagoya-u.ac.jp

Supplementary Figure S1. The sizes of left diaphragm and innervated NMJ of left diaphragm in wild-type (WT), *Rspo2*$^{-/-}$, MCK-*RSPO2/Rspo2*$^{-/-}$, and VAChT-*RSPO2/Rspo2*$^{-/-}$ embryos at E18.5

(A) Representative images of the left hemi-diaphragms at E18.5 stained with α-bungarotoxin (red) and anti-peripherin antibody (green). **(B)** Ratios of innervated NMJs in the left diaphragm at E18.5. AChR-positive areas (red in **A**) overlapping with peripherin-positive areas (green in **A**) were defined as innervated NMJs (*n* > 50 NMJs), which were divided by the number of AChR-positive areas (red in **A**) to calculate the ratio of innervated NMJs. **(C)** Schematic of the left diaphragm indicating the width, length, thickness and area. **(D-G)** Morphometric analysis was performed on microscopic images of the left diaphragms of WT, *Rspo2*$^{-/-}$, MCK-*RSPO2/Rspo2*$^{-/-}$, and VAChT-*RSPO2/Rspo2*$^{-/-}$ mice. Mean ± SEM (*n* = 3 left diaphragms in each group) are indicated. **(B, D, E, F, and G)** No statistical significance (n.s.) was observed by one-way ANOVA.

Supplementary Figure S2. Enlarged electron micrographs of the NMJs of the left diaphragms indicated in Fig. 5A.

Representative electron micrographs of the left diaphragm NMJs of wild-type (WT), *Rspo2*$^{-/-}$, MCK-*RSPO2/Rspo2*$^{-/-}$, and VAChT-*RSPO2/Rspo2*$^{-/-}$ mice at E18.5. Areas in red rectangles in Fig. 5A are enlarged. Red arrows indicate representative, but not all, synaptic vesicles. White upper arrowheads indicate postsynaptic folds. Blinded morphometric measurements are shown in Fig. 5B-H. SV, synaptic vesicles. Scale bar = 500 nm.

Supplementary Table S1. Sequences of oligonucleotides for QuikChange site-directed mutagenesis (SDM) and for PCR

Primers	Primer Sequence 5'-3'
NotI-CMV-F for SDM	GTGCCAAGCTGCGGCCGCGATCTATACA
NotI-CMV-R for SDM	TGTATAGATCGCGGCCGCAGCTTGGCAC
EcoRV-polyA-F for SDM	CCCTGTCCTTGATATCCTGATTTTAA
EcoRV-polyA-R for SDM	TTAAAATCAGGATATCAAGGACAGGG
Rspo2-HindIII-F for cloning	AACCAAGCTTATGCGTTTTTGCCTCTTCTC
Rspo2-XbaI-R for cloning	AACCTCTAGATTGGTTCACTCTGTCTGTAG
MCK-NotI-F for cloning	ATAAGCGGCCGCTCTCCCCTCAGCATTCCTTC
MCK-HindIII-R for cloning	AATAAAGCTTGTGACCCGGGGGCAGCCCCT
VAChT-NotI-F for cloning	AAAAGCGGCCGCTGGAAACATCTCGGAAGCG
VAChT-HindIII-R for cloning	TTAAAAGCTTTGTACAGGCATCTTTGGGGG
Rspo2 shared-F for genotyping	GATCTACACCTGGTGCTGGGGC
Rspo2 wild-type-R for genotyping	CCATTCAGGCTGCGCAACTGTTGG
Rspo2 mutant-R for genotyping	GCATCTGGACGGTCGGGCTGGG
MCK promoter-F for genotyping	AAGCTCATCTGCTCTCAGGGG
VAChT promoter-F for genotyping	AAACCAAGGTGCTCTAGTGC
Rspo2-R for genotyping	GTTTGGTTTCCAGACCCCAT

Restriction sites are underlined.

Supplementary Table S2. Sequences of real-time RT-PCR primers

Genes	Forward primers	Reverse primers
Rspo2	GGTTTTGCACCGTTAGATGAGA	GTTCTGGTTTCCAGACCCCAT
Lgr5	GAGCGTTCGTAGGCAACCCTTCTC	GGTGGCAGTTCCTGTCAAGTGAGG
Musk	CCTACCCTCAGCCCGAGATTTCTTG	GCCATCATCACTGTCTTCCACGCTC
Lrp4	CCCTGCAAGTGAAGATGAAA	TTCAAGAACTGCGATTCTGG
Ctnnb1	TTAAACTCCTGCACCCACCAT	AGGGCAAGGTTTCGAATCAA
Myc	TGTCCATTCAAGCAGACGAG	GCATTTTAATTCCAGCGCAT
Axin2	GAAACTGGCAAGTGTCCACG	CGCAGGCAAATTCGTCACTC
Myod	CCGCCTGAGCAAAGTGAATG	GCGGTCCAGGTGCGTAGAA
Pax7	AGAGGACGACGAGGAAGGAG	GGGAGGTCGGGTTCTGATT
Chat	TGCAACACCTGGTACCTGAA	GCAGGGCTAGAGTTGACTGG
Agrn	CAGTGGGGGACCTAGAAACA	GCCATGTAGTCTGCACGTTCT

Supplementary Figure S1

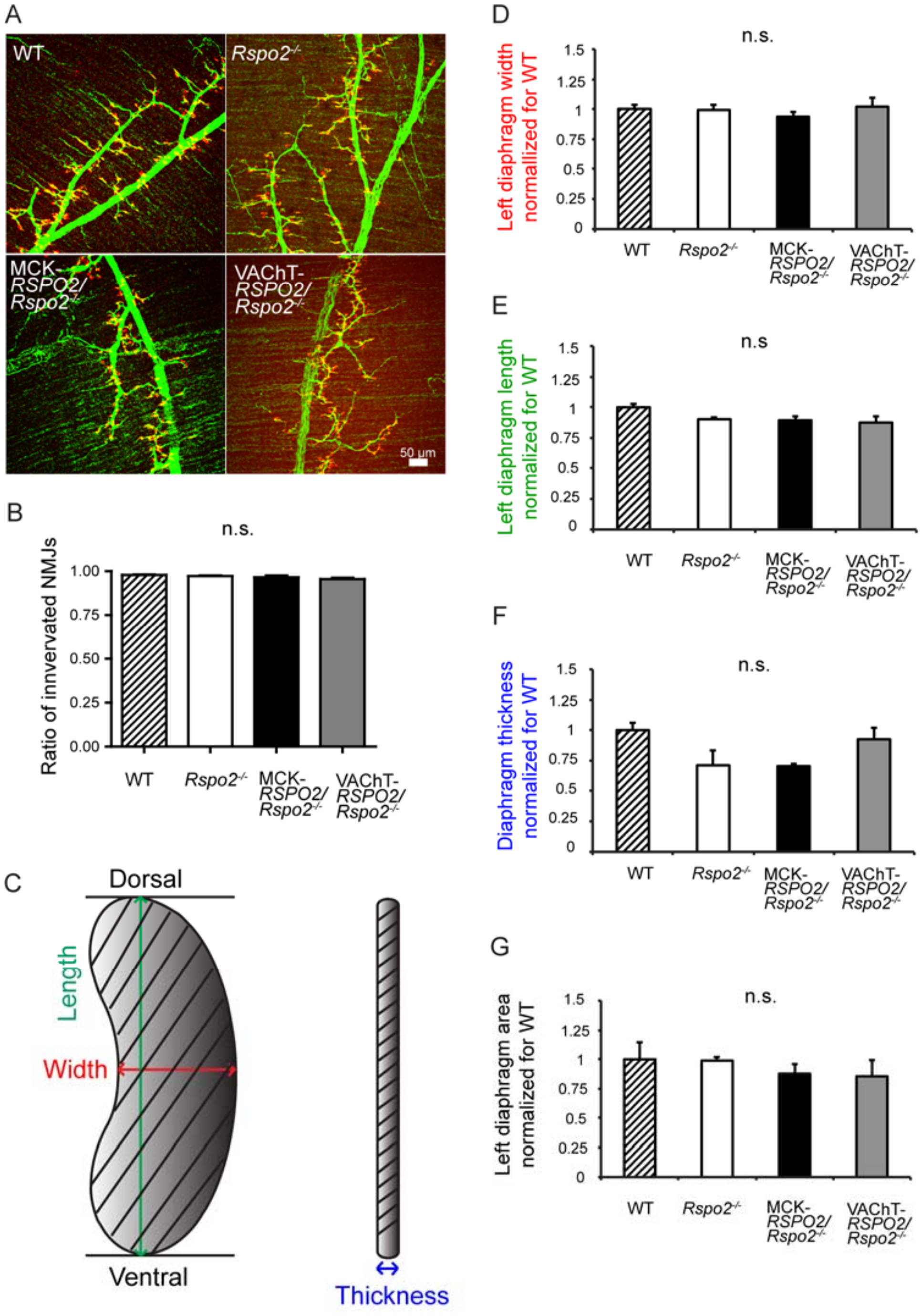

Supplemetary Figure S2

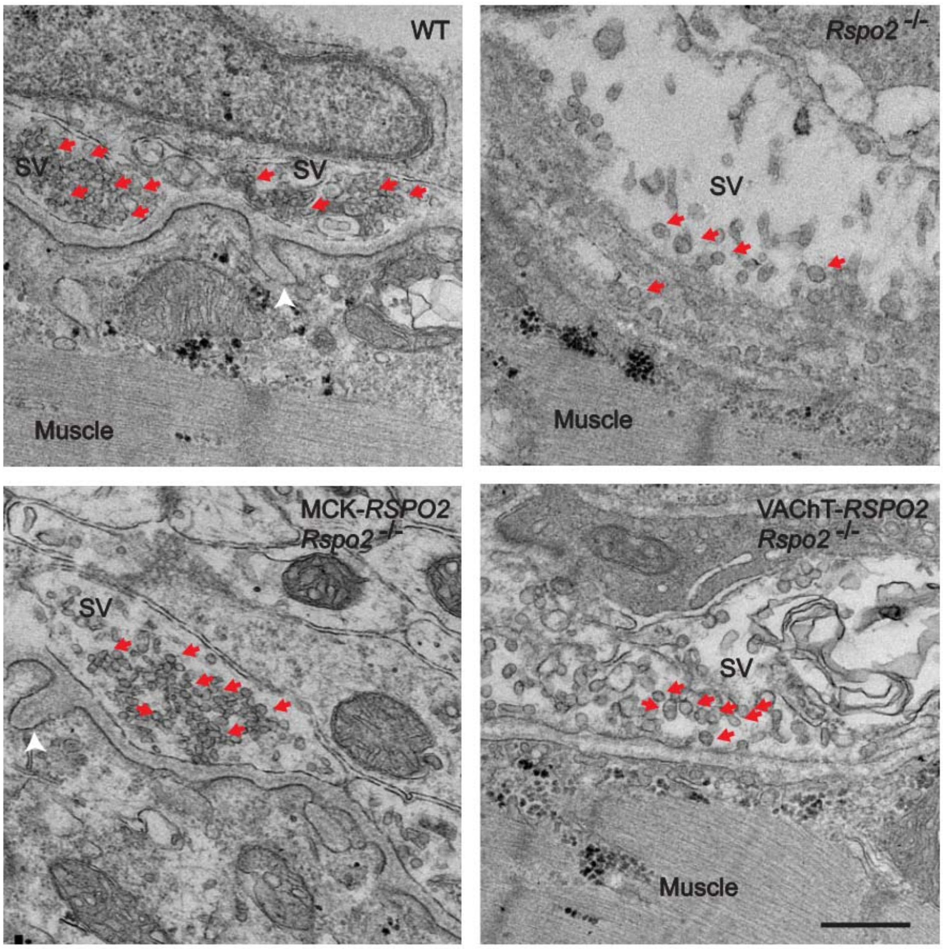

Supplement: Supplementary file 1 — Supplementary Information [file 41598_2018_31949_MOESM1_ESM.pdf]
